# Heart Transplantation for Peripartum Cardiomyopathy: A Single-Center Experience

**DOI:** 10.5935/abc.20180014

**Published:** 2018-02

**Authors:** Nadia Bouabdallaoui, Pierre Demondion, Sylvestre Maréchaux, Shaida Varnous, Guillaume Lebreton, Frédéric Mouquet, Pascal Leprince

**Affiliations:** 1 Department of Cardiac Surgery, La Pitié Salpêtrière, Assistance Publique des Hôpitaux de Paris; Université Pierre et Marie Curie-Paris 6, France; 2 GCS-Groupement des Hôpitaux de l’Institut Catholique de Lille, Cardiology Department and Heart Valve Center, Faculté Libre de Médecine/Université Catholique de Lille, France; 3 Service de Cardiologie, Pôle Cardio-vasculaire et Pulmonaire, Hôpital Cardiologique, CHRU Lille, Lille Cedex, France

**Keywords:** Heart Failure, Cardiomyopathies / mortality, Peripartum Period, Heart Transplantation, Graft Rejection. / mortality

## Abstract

**Background:**

Peripartum cardiomyopathy is an idiopathic disorder defined by the occurrence of acute heart failure during late pregnancy or post-partum period in the absence of any other definable cause. Its clinical course is variable and severe cases might require heart transplantation.

**Objective:**

To investigate long-term outcomes after heart transplantation (HT) for peripartum cardiomyopathy (PPCM).

**Methods:**

Out of a single-center series of 1938 HT, 14 HT were performed for PPCM. We evaluated clinical characteristics, transplant-related complications, and long-term outcomes, in comparison with 28 sex-matched controls. Primary endpoint was death from any cause; secondary endpoints were transplant-related complications (rejection, infection, cardiac allograft vasculopathy). A value of p < 0.05 was considered of statistical significance.

**Results:**

PPCM patients and matched controls were comparable for most variables (all p values > 0.05), except for a higher use of inotropes at the time of HT in PPCM group (p = 0.03). During a median follow-up of 7.7 years, 16 patients died, 3 (21.5%) in PPCM group and 13 (46.5%) in control group. Mortality was significantly lower in PPCM group (p = 0.03). No significant difference was found in terms of transplant-related complications (p > 0.05).

**Conclusions:**

Long-term outcomes following HT for PPCM are favorable. Heart transplantation is a valuable option for PPCM patients who did not recover significantly under medical treatment.

## Introduction

Peripartum cardiomyopathy (PPCM) is defined by the occurrence of acute heart failure (HF) during late pregnancy or post-partum period in the absence of any other definable cause or prior heart disease. Diagnostic criteria have recently been revised by the ESC Working Group on PPCM.^1^ Disease incidence shows ethnic variations, with a greater prevalence among African women.^2^ A deleterious combination of “anti angiogenic signaling excess” and “oxidative stress-prolactin axis” toward the end of pregnancy is suggested as key element in the pathophysiology of the disease.^3^ Beside conventional treatment of HF,^4^ targeted therapies including pharmacological prolactin blockade are being investigated.^5^ Although half of patients will fully recover left ventricular systolic function, the clinical course of PPCM is highly variable.^6,7^ Data from the Investigations of Pregnancy-Associated Cardiomyopathy (IPAC) recently assessed a 6% rate of death, heart transplantation, and left ventricular assist device (LVAD) implantation at 1 year in PPCM patients and more than 20% rate of persistent left ventricular (LV) dysfunction.^6^ Baseline LVEF < 30%, baseline LV end-diastolic diameter (LVEDD) > 60mm, black ethnicity and post-partum diagnosis were correlated with poor prognosis.^6^ Up to 10% of PPCM patients will require heart transplantation according to literature data.^6,8-10^ Post-transplant prognosis for PPCM patients is at present still contradictory.^11-14^ A higher incidence of rejection has been reported, particularly during the first year following transplantation, along with a lower graft survival.^13,14^ Heart transplantation (HT) is however considered as a valuable option for PPCM patients presenting with HF unresponsive to maximal conventional treatment. The aim of this study is to compare all-cause mortality and transplant-related complications after HT for PPCM.

## Methods

This is a retrospective single-center non-interventional study. Primary endpoint was all-cause mortality following heart transplantation (HT). Secondary endpoint was outcomes after HT including transplant-related complications (rejection, infection, cardiac allograft vasculopathy). All patients had single-center management with a consistent approach at both surgical and medical levels.

### Patient population

A total of 1938 patients from whom 368 females were transplanted for severe HF in our institution. Fourteen patients met diagnosis criteria of PPCM. All our PPCM cases were ascertained with the most recent definition of the disease.^1^ An extensive work-up was performed retrospectively for each patient to exclude other causes of HF. Twenty-eight age-matched female patients who underwent HT during the same period for other causes served as controls. Each PPCM patient was matched to two female control patients depending on their age at the time of transplantation (± 5 years) and on the era of transplantation (± 6 months). Survival was assessed until last follow-up. Demographics, pre- and post-transplant data were retrospectively collected from our institution’s computerized medical charts. Information on follow-up was obtained retrospectively by direct patient interview for those who were still alive at the time of data collection. As this was an observational study, our institutional ethics board was not involved.

### Post-transplant course

All patients had a similar post-transplant follow-up protocol. Endomyocardial biopsies were routinely performed during the first two years following HT, then, less frequently (every 6 months for years 2 to 5, then every year beyond 5th year), unless clinical indication. Coronary angiography was first performed at one-year post transplant then every two years if normal. We considered arbitrarily graft rejection as present or non-present, regardless of its type (antibody-mediated or cell-mediated rejection) and severity. The diagnosis of cell mediated rejection was based on Stanford grading system until 1990,^15^ then, on the International Society for Heart and Lung Transplantation nomenclature (ISHLT;^16^). The ISHLT Guidelines on Antibodies-mediated rejection (AMR) were used for the definition of AMR rejections.^17,18^ We considered rejection as “characterized” in the following situations: All cell-mediated rejections of grade > or = to 1A/1R; All proven antibodies mediated rejection regardless of grade; All symptomatic rejections i.e. with hemodynamic compromise or LV dysfunction.^19^ All characterized rejections triggered therapeutic interventions.

Cardiac Allograft Vasculopathy (CAV) was considered in the setting of any angiographic evidence of coronary artery stenosis regardless of the need of specific treatment.^20^ Infections were defined as any episode requiring hospitalization or intravenous treatment, including cytomegalovirus (CMV) infections.

Immunosuppressive therapy and rejection treatments varied over time. Induction therapy involved intravenous methylprednisolone, and rabbit anti-thymocyte globulin from 1986 to 2000; and antithymocyte globulin or Basiliximab since 2000. Long-term prophylactic immunosuppressive therapy was based on calcineurin inhibitors (mostly cyclosporine), azathioprine and long-term oral corticosteroids from 1986 to 2000; and calcineurin inhibitors (cyclosporine or tacrolimus), mycophenolate mofetil and oral corticosteroids since 2000. Everolimus was not routinely used upon the study population. Of note, none of the patients in PPCM group received Bromocriptine.

### Statistical considerations and analysis

Data are presented as the mean ± standard deviation, unless otherwise specified. Comparisons between groups for continuous variables were performed using the Student t-test or the Mann Whitney U test as appropriate. The chi-square or the Fisher exact tests were used for categorical variables as appropriate. The duration of follow up was computed using reverse the Kaplan Meier method. Survival was defined as being alive at the cut-off date for our study without the need of a retransplantation. Kaplan-Meier survival curves were constructed for the two groups and compared using the log rank test. A value of p < 0.05 was considered of statistical significance. All analyses were conducted with the use of SPSS 18.0 software (Chicago, Illinois).

## Results

### Pre-transplant characteristics

Pre-transplant characteristics are summarized in Tables 1 and 2. Patients in control group were transplanted for: idiopathic dilated (n = 10, 36%), ischemic (n = 8, 28.5%), congenital (n = 1, 3.5%), restrictive (n = 2, 7.1%), valvular (n = 2, 7.1%), and anthracyclines-induced (n = 3, 10.7%) cardiomyopathies or myocarditis (n = 2, 7.1%). There were significantly more patients requiring inotropes in PPCM group (n= 9, 64% in PPCM patients vs. n = 8, 28% in controls, p = 0.03). Patients requiring hemodynamic support were indiscriminately those recently diagnosed with PPCM and readily presenting with cardiogenic shock (n = 4/9), but also those with long time known PPCM and gradually progressing to end-stage heart failure (n = 5/9). Conversely, in control group, patients requiring inotropic support were more often those who were recently (< 1year) diagnosed with HF.

**Table 1:** General characteristics of PPCM patients

PPCM patients	Time from diagnosis to HT	Time on waiting list	Age at the time of HT	LVEF (%)	Inotropes	IABP	ECMO (P + C)	VAD	Cross-match
1	19 yrs	1 mth	49	30	Y	N	N	N	N
2	2 yrs	18 mths	30	15	N	N	N	N	N
3	8 yrs	< 1 mth	36	25	Y	N	N	N	N
4	10 mths	1 mth	39	25	N	N	N	N	N
5	5 mths	< 1 mth	35	10	Y	N	N	Y	N
6	3 mths	< 1 mth	35	23	N	N	N	N	N
7	13 yrs	< 1 mth	44	20	Y	N	N	N	N
8	1 mth	< 1 mth	33	14	Y	N	N	N	NA
9	4 mths	1 mth	29	15	Y	Y	Y	N	N
10	4 yrs	9 mths	34	32	Y	N	Y	Y	N
11	15 yrs	2 mths	47	25	N	N	N	N	N
12	1 yr	< 1 mth	27	10	N	N	N	N	NA
13	9 mths	< 1 mth	37	25	Y	N	N	N	NA
14	1 yr	2 mths	39	35	Y	N	N	N	N

LVEF: Left Ventricle Ejection Fraction; IABP: intra-aortic balloon counterpulsation; ECMO (P+C): Extra Corporeal Membrane Oxygenation (Peripheral + Central); VAD: Ventricular Assist Device; Y: Yes; N: No; NA: Not applicable; yr: year; m: month.

**Table 2:** Pre-transplant characteristics in PPCM group and control subjects

Variable	PPCM group (n = 14)	Control group (n = 28)	p
Age at the time of HT, years	36.7 ± 6.5	38.4 ± 8.5	p = 0.4
Previous pregnancies	100% (n = 14)	50% (n = 14)	p = 0.3
Smoker	21% (n = 3)	42.8% (n = 12)	p = 0.1
Hypertension	7% (n = 1)	7% (n = 2)	p = 0.7
Beta-blockers	50% (n = 7)	42.8% (n = 12)	p = 0.5
ACE inhibitors	50% (n = 7)	75% (n = 21)	p = 0.6
Time on waiting list, months	2.4 ± 5	3.8 ± 5	p = 0.1
LVEF (%)	22 ± 8	24 ± 14	p = 0.9
Inotropes	64% (n = 9)	28.57% (n = 8)	p = 0.03
IABP	7% (n = 1)	7% (n = 2)	p = 0.7
ECMO	14% (n = 2)	25% (n = 7)	p = 0.5
VAD	14% (n = 2)	7% (n = 2)	p = 0.4

PPCM: peripartum cardiomyopathy; LVEF: Left Ventricle Ejection Fraction; RV: Right Ventricle; IABP: intra-aortic balloon counter pulsation; ECMO (P+C): Extra Corporeal Membrane Oxygenation (Peripheral + Central); VAD: Ventricular Assist Device. (Comparisons between groups for continuous variables were performed using the Student t test or the Mann Whitney U test as appropriate).

We found no significant difference considering African descent; the time spent on the transplant waiting list; right ventricular dysfunction; and HF severity at the time of diagnosis. No significant difference in HF treatment was noticed particularly in terms of ACE inhibitors or beta-blockers administration, and cardiac resynchronization therapy (CRT) / internal cardioverter defibrillator (ICD) implantation rates.

Regarding mechanical circulatory support (MCS) indication, no significant difference was observed. In PPCM group, one patient underwent intra-aortic balloon counterpulsation (IABP), two peripheral Extra-Corporeal Membrane Oxygenation (ECMO), one long-term Ventricular Assist Devices, and one CardioWest Total Artificial Heart implantation. In control group, two patients underwent IABP, seven peripheral or central ECMO, and two long term VADs.

### Graft Characteristics and Immunosuppressive treatments

Graft characteristics were similar in the two groups. Mean ischemic time duration was 159 ± 12 minutes in PPCM group vs. 178 ± 13 minutes in control group. Mean age donor was 45 years for PPCM recipients and 46 years for controls. We observed no significant difference in terms of sex mismatch. As the patients were matched for transplantation period, there was no difference in immunosuppressive regimen.

### Patients outcomes

**Figure 1:**
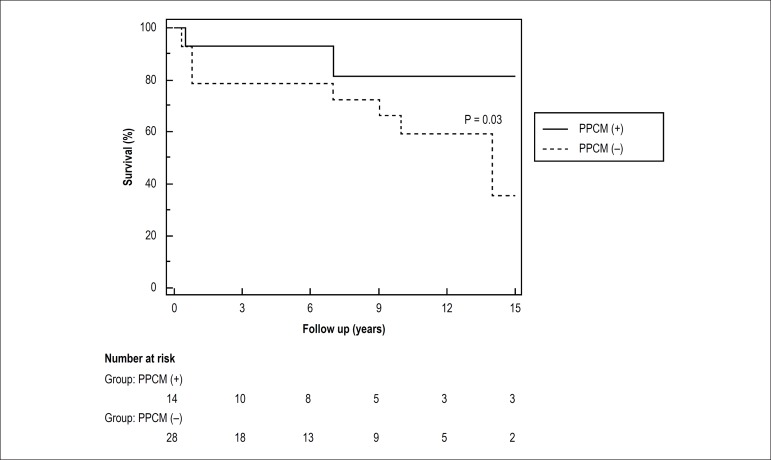
Long-term survival after heart transplantation, PPCM group (PPCM (+)) and control patients (PPCM (-)). PPCM: peripartum cardiomyopathy.

During a median follow-up of 7.7 years, 16 patients died, 3 (21.5%) in PPCM group and 13 (46.5%) in control group. Mortality was significantly lower in PPCM group (p = 0.03, Figure 1). Causes of death are shown in Table 3. Major causes of one-year mortality after HT were rejection, hemorrhagic complications and infections; major causes of long-term mortality (> 1 year) after HT were rejection, CAV, and infections. Both early and late rejection rates were similar in both groups (p = 0.5 and 0.6 respectively). PPCM patients had a similar incidence of infections including cytomegalovirus (CMV) infections compared with control population (p = 0.07). Two patients from control group died within the first year following transplantation from septic shock, none in PPCM group. One more patient in control group died from septic shock > 1 year post transplant, none in PPCM group. PPCM patients had a similar risk of CAV compared with control group (p = 0.4). Pathological study of explanted hearts did not reveal any specific lesion.

**Table 3:** Transplant-related complications and causes of Death

Transplant-related complications	PPCM group (n = 14)	Control group (n = 28)	p
**Rejection:**			
Treated rejections < 1-year post transplant	50% (n = 7)	50% (n = 14)	p = 0.5
Treated rejections > 1-year post transplant	71% (n = 10)	50% (n = 14)	p = 0.6
Infection rate	35.7% (n = 5)	64.3% (n = 18)	p = 0.07
CAV	50% (n = 7)	35.7% (n = 10)	p = 0.4
Death: Early all-cause mortality (< 1 year)	7% (n = 1)	21.4% (n = 6)	p = 0.06
Rejection	n = 0	n = 1	
Infection	n = 0	n = 2	
CAV	n = 0	n = 0	
Hemorrhagic complications	n = 1	n = 2	
Thromboembolic complications	n = 0	n = 1	
Death: Late all-cause mortality (> 1 year)	21.4% (n = 3)	46.4% (n = 13)	p = 0.07
Rejection	n = 1	n = 2	
Infection	n = 0	n = 3	
CAV	n = 1	n = 4	
Hemorrhagic complications	n = 1	n = 2	
Thromboembolic complications	n = 0	n = 1	
Neoplasia	n = 0	n = 1	
Unknown	n = 1	n = 0	

CAV: Cardiac Allograft Vasculopathy (Comparisons between groups for continuous variables were performed using the Student t-test or the Mann Whitney U test as appropriate).

## Discussion

In this retrospective single-center study, we assessed post-transplant outcomes in a population of patients transplanted for severe HF in the setting of peripartum cardiomyopathy. Median follow-up was 7.7 years. We demonstrate upon our population that post-transplant mortality is significantly lower in patients transplanted for PPCM. Patients transplanted for PPCM did not display a significantly higher rate of transplant-related complications compared with control subjects matched for age and transplantation period.

### Pre-transplant characteristics

In the pre-transplant setting, we significantly used more inotropes at the time of HT in PPCM patients compared with control subjects. The frequent need of medical intensive cardiovascular support in PPCM patients awaiting heart transplantation has also been demonstrated by others.^13^ Importantly, potential deleterious cellular alterations related to Dobutamine have recently been pointed in PPCM patients,^21^ and recent guidelines recommend a cautious use of inotropes for critically-ill PPCM patients.^22^

Data related to MCS in the management of PPCM patients are scarce.^23,24^ It seems however that MCS is an option for patients who deteriorate despite maximal therapy, in a strategy of bridge to transplantation or to recovery.^6,22-25^ Noticeably, one major concern in the setting of long-term MCS in PPCM patients relates to a possibly higher risk of thrombotic complications in a prothrombotic condition such as the peripartum period.^26^

Medical management of HF might be considered as non-optimal in our population, particularly among PPCM patients, as only one half received beta-blockers and ACE inhibitors. Importantly, under-treated patients were, in both groups, those requiring inotropic and mechanical circulatory support.

Seven percent (7%) of patients had CRT/ICD implantation. Recent data suggest that CRT is crucial in the management of PPCM patients presenting with persistent systolic dysfunction. It has indeed been demonstrated a rapid and significant LV recovery under CRT in PPCM patients with severe systolic dysfunction despite optimal medical therapy.^27^

### Patients Outcomes after Heart Transplantation

We assessed post-transplant outcomes in patients transplanted for PPCM. Again, we demonstrated a significantly lower post-transplant all-cause mortality in patients transplanted for PPCM, with a similar rate of transplant-related complications as compared with control subjects. Data on long-term outcomes after HT for PPCM are contradictory, reporting either favorable outcomes,^11^ or higher rejection rates and poorer outcomes.^12-14^ Current practice is however favorable to HT for PPCM. As we did, a long-term survey of a small cohort of patients transplanted for PPCM has also shown favorable outcomes.^23^

### Limitations

The major limitation of our study is the small number of patients, prohibiting definitive conclusions. We arbitrarily adjudicated rejection in a binary way (present: yes, or no), which might therefore be considered as simplistic and of limited value.

## Conclusion

We assessed long-term post-transplant outcomes in the setting of PPCM. Upon the studied population, we demonstrate a significantly lower long-term post-transplant mortality in patients transplanted for PPCM, with a similar rate of transplant-related complications as compared with control subjects. We show that heart transplantation for PPCM patients who did not significantly recover under maximal medical treatment remains appropriate. The overall impact of heart transplantation for PPCM is yet to be determined at a larger scale in well characterized population.
